# Imaging the role of blood–brain barrier disruption in normal cognitive ageing

**DOI:** 10.1007/s11357-020-00282-1

**Published:** 2020-10-06

**Authors:** Inge C. M. Verheggen, Joost J. A. de Jong, Martin P. J. van Boxtel, Alida A. Postma, Jacobus F. A. Jansen, Frans R. J. Verhey, Walter H. Backes

**Affiliations:** 1 Department of Psychiatry and Neuropsychology, Maastricht University, P.O. Box 616, 6200 MD Maastricht, The Netherlands; 2 School for Mental Health and Neuroscience (MHeNs), Maastricht University, Maastricht, The Netherlands; 3 Alzheimer Center Limburg, Maastricht, The Netherlands; 4 Department of Radiology and Nuclear Medicine, Maastricht University Medical Center, Maastricht, The Netherlands; 5 Department of Electrical Engineering, Eindhoven University of Technology, Eindhoven, The Netherlands; 6 School for Cardiovascular Research Institute Maastricht (CARIM), Maastricht University, Maastricht, The Netherlands

**Keywords:** Blood–brain barrier, Cognitive ageing, Dynamic contrast–enhanced MRI, Cerebrovascular dysfunction, Successful ageing

## Abstract

To investigate whether blood–brain barrier (BBB) disruption is a potential mechanism of usual age-related cognitive decline, we conducted dynamic contrast–enhanced (DCE) MRI to measure BBB leakage in a healthy sample, and investigated the association with longitudinal cognitive decline. In a sample of neurologically and cognitively healthy, older individuals, BBB leakage rate in the white and grey matter and hippocampus was measured using DCE MRI with pharmacokinetic modelling. Regression analysis was performed to investigate whether the leakage rate was associated with decline in cognitive performance (memory encoding, memory retrieval, executive functioning and processing speed) over 12 years. White and grey matter BBB leakages were significantly associated with decline in memory retrieval. No significant relations were found between hippocampal BBB leakage and cognitive performance. BBB disruption already being associated with usual cognitive ageing, supports that this neurovascular alteration is a possible explanation for the cognitive decline inherent to the ageing process. More insight into BBB leakage during the normal ageing process could improve estimation and interpretation of leakage rate in pathological conditions. The current results might also stimulate the search for strategies to maintain BBB integrity and help increase the proportion people experiencing successful ageing. Netherlands Trial Register number: NL6358, date of registration: 2017-03-24.

## Introduction

Over the last years, population demographics have been shifting towards a larger proportion of older individuals, due to a combination of decreasing birth rates and increasing life expectancy [1]. With a larger older population, determining which factors could lead towards successful ageing has become an important scientific objective even more. Besides the absence of disease and disability, an important component of successful ageing is the maintenance of cognitive functioning [2]. An ageing population is increasingly confronted with age-related neurobiological changes, and such changes in brain structure and function are often associated with changes in cognition [3, 4]. However, ageing does not necessarily lead to cognitive impairment, as some people age without considerable cognitive setback, while others show substantial cognitive decline [5]. Investigating what neurobiological changes lead to age-related cognitive decline and hinder successful ageing is becoming increasingly important [4, 6].

Age has a large impact on the cerebrovascular system and is a major risk factor for cerebrovascular damage [7, 8]. Cerebrovascular disease is therefore a highly prevalent age-related condition [9]. Moreover, cerebrovascular disease appears to affect cognition even in elderly in the normal ageing spectrum [10]. The blood–brain barrier (BBB) is part of the cerebrovascular system and was shown to be vulnerable to age-related changes [9, 11, 12]. Recently, higher BBB permeability has been linked to an increasing number of age-related neurological disorders [8, 13, 14]. Previous studies were even able to find an association between cognitive impairment inherent to these conditions and BBB disruption [8, 15–17].

At the level of the microvascular network, specialized cells work together in a neurovascular unit (NVU) to maintain cerebral blood flow (CBF), oxygen delivery and energy supply [18]. The NVU consists of vascular cells (i.e. endothelial cells), glial cells (i.e. astrocytes) and neurons [18, 19]. Interaction between vascular cells and neurons in the NVU creates hemodynamic coupling, allowing an increase in neuronal activation to be followed by an appropriate CBF response [20, 21]. The endothelial cells are connected by tight junctions to create a strong barrier forming the basis of the BBB [18]. The BBB separates blood plasma from brain tissue and regulates the delivery of energy metabolites and nutrients to the neurons, while preventing neurotoxins from entering the brain.

The endothelium allows small molecules as oxygen and carbon dioxide to rapidly and freely diffuse across the BBB, which is essential for brain metabolism and pH regulation [22]. Small, lipophilic molecules can also cross the BBB [23]. As for the rest, the endothelium contains specialized transport systems using active efflux, carrier-mediated transport and receptor-mediated transport to enable nutrients to pass from blood plasma to brain tissue and to remove waste products from the brain [22, 24]. The BBB thereby protects the brain from ion fluctuations that occur in the blood after a meal or exercise and ensures homeostasis is maintained, which is essential for neuronal and synaptic functioning [25, 26].

Neurovascular dysfunction has been suggested to initiate the cascade leading to neurodegeneration and AD [18, 25, 27]. Neurovascular dysfunction involves uncoupling of the NVU, which can lead reduction and dysregulation of CBF and disruption of the BBB [28, 29]. A recent hypothesis for instance states that depletion of the nicotinamide adenine dinucleotide (NAD^+^) substrate can lead to endothelial dysfunction, which impairs the integrity of the cerebral microcirculation [30]. Both CBF and BBB disruption impair the supply of oxygen and nutrients to the neurons. As a result, hypoxia and inflammation may occur and trigger subsequent pathological processes, which make the brain vulnerable to neuronal dysfunction, and eventually neurodegeneration [8, 31, 32]. Moreover, BBB disruption reduces the clearance of interstitial solutes from the brain and could lead to accumulation of toxic waste products, such as the amyloid-β protein (Aβ) in case of AD [33]. In turn, Aβ can further impair neurovascular functioning, including CBF regulation and BBB integrity. These neurovascular alterations occur even before the formation of Aβ plaques, suggesting that they are an early event in the pathological cascade [34–36], which is supported by several imaging studies demonstrating early BBB disruption in the development of AD [15, 37–39]. Studies have even shown that carrying the ε4 allelle of the apolipoprotein E gene (APOE4), indicating genetic susceptiblility to AD [40], is associated with accelerated BBB disruption [41–43].

Thus, BBB disruption is considered as an early event in the pathological cascade possibly leading to neurodegeneration [15, 38]. Early BBB leakage was also associated with global cognitive functioning, as leakage rates in individuals with early AD and healthy age–matched controls correlated significantly with cognitive function (Mini-Mental State Examination, MMSE [44]) [15]. Moreover, individuals with early cognitive dysfunction, not accounted for by a neurological disorder, demonstrated increased BBB disruption independent of Aβ or tau [17]. This suggests that BBB breakdown could already be present in those who experience age-related cognitive decline, even without having a neurological disorder. A recent study demonstrated that BBB disruption is not only more prominent in APOE4 carriers, but this effect was stronger in cognitively impaired individuals, which demonstrates that BBB disruption is involved in APOE4-related cognitive decline [45]. BBB disruption might be a contributing mechanism to cognitive ageing.

Interest in BBB disruption is increasing due to recent discoveries about its role in brain pathology, but also due to recent advances made in the development of magnetic resonance imaging (MRI) techniques to measure permeability [31, 46]. One of the most promising techniques is dynamic contrast–enhanced (DCE) MRI, which measures contrast agent leakage from the blood plasma to the brain interstitial space over time, and allows the detection of subtle leakage values, for instance in dementia [47]. Using DCE MRI has made it possible to detect increased BBB leakage in mild cognitive impairment (MCI), the transition stage to AD [16] and small vessel disease [48], the most important cause of vascular dementia [49]. Increased BBB leakage at older ages was even found in healthy individuals [50]. However, whether BBB leakage is associated with variation in usual age-related cognitive decline remains to be investigated.

The current study used dual-time resolution DCE MRI to investigate whether BBB breakdown is already associated with cognitive decline in cognitively and neurologically healthy, older individuals. We hypothesised that BBB leakage would be significantly associated with usual age-related cognitive decline, which would support BBB disruption as contributing mechanism to cognitive ageing.

## Methods

### Participants

Fifty-seven participants (mean age, 66 years; age range, 47–91 years) were recruited from the Maastricht Aging Study (MAAS) [51]. Cognitively and neurologically healthy participants were selected, based on the following criteria: MMSE [44] score ≥ 25; Disabilities Assessment of Dementia (DAD [52]) score ≥ 90%; as reported in their medical history: no diagnosis of dementia, prodromal dementia, MCI, or any other psychiatric or neurological condition, no large structural brain abnormalities, brain surgery or brain trauma, and no cognitive impairment due to substance abuse; no contraindications for MRI scanning or gadolinium-based contrast agent (sufficiently functioning kidneys with glomerular filtration rate (eGFR) > 30 mL/min).

### Cognitive decline

Participants performed a short battery of standardized cognitive tests. Memory function was measured using the verbal learning test (VLT [53]), in which the immediate recall score gives an indication of short-term episodic memory and learning, and the delayed recall score is considered to be a measure of long-term episodic memory. Processing speed was measured with the letter-digit substitution test (LDST [54]). Executive functioning was measured with the Stroop colour-word test [55], in which the interference score is considered to be a measure of inhibition.

Cognitive decline was calculated by subtracting the participants’ current score from their previous score in the last measure of MAAS approximately 12 years ago, so that a larger difference score would correspond to more cognitive decline. The interference score of the Stroop test was obtained using the time participants needed to complete the task and was the only test in which a larger difference score indicated less decline. The difference score of Stroop interference was reversed by multiplying difference scores with − 1, so that a larger score would correspond to more cognitive decline.

### MRI acquisition

Images were acquired on a 3-Tesla MRI scanner (Achieva TX, Philips Healthcare, Best, The Netherlands) with a 32-channel head coil. The imaging protocol included a 3D T1–weighted sequence for anatomical reference, a 3D T2–weighted fluid attenuation inversion recovery (FLAIR) sequence for brain segmentation and a dual-time resolution dynamic contrast–enhanced (DCE) sequence for leakage calculations.

The fast sequence of the dual-time resolution DCE MRI had a dynamic scan interval of 3.2 s and consisted of 29 volumes with a voxel size of 2 × 2 × 5 mm. During the fast sequence, the gadolinium-containing contrast agent (0.1 mmol/kg gadobutrol, Gadavist®, Bayer AG, Leverkusen, Germany) was injected in the antecubital vein (injection rate 3 mL/s, 20 mL saline flush). After the rapid spread over the vasculature, a slow sequence was applied for leakage into the brain tissue. The slow sequence had a dynamic scan interval of 30.5 s and consisted of 30 volumes with a voxel size of 1 × 1 × 2 mm. Conversion of tissue signal intensity to contrast agent concentration was enabled by T1-mapping.

### Structural brain characteristics and segmentation

T2-FLAIR images were also used to obtain scores on the Fazekas scale [56] for white matter hyperintensity (WMH) load, and T1-weighted images to obtain scores on the global cortical atrophy (GCA) scale [57] and the medial temporal lobe atrophy (MTA) scale [58]. Categorical scores on these visual rating scales were assigned by experienced neuroradiologists (A.A.P and W.M.P.).

WMH volume (cm^3^) was quantified using the in-house developed, semi-automated segmentation tool GIANT [59] and corrected for intracranial volume, as volumetric measures may be confounded by their correlation with head size [60].

Automated brain segmentations were created using FreeSurfer software (version 6.0.0 [61]), after which these segmentations were visually checked and manually adjusted (I.C.M.V.). From the FreeSurfer output, cortical thickness averaged over the whole brain (mm) and hippocampal volume averaged over the hemispheres (cm^3^) were obtained, after which hippocampal volume was corrected for intracranial volume. For our analyses, total white matter, total grey matter (cortical and deep grey matter and hippocampus) and the hippocampus separately were extracted to create tissue masks [61].

### DCE MRI processing

Both the fast and slow DCE MRI were motion corrected and a reference image was created using the average of the pre-contrast images in each sequence. The fast DCE MRI data was co-registered on the slow DCE MRI, and the reference slow DCE MRI was registered on the T1-weighted images for anatomical reference. Ultimately, the inverse of the obtained transformation matrix was used to transform the T1-weighted data to slow DCE space.

An individual vascular input function (VIF) was obtained for each participant by manually (I.C.M.V.) selecting at least 20 voxels in the superior sagittal sinus. The tissue signal in the brain ROIs was converted to contrast concentration by assuming a linear relationship and using the tissue relaxation time from the T_10_-map, while the blood plasma signal in the VIF was converted to contrast concentration using in vitro data [62] (diluted MnCl_2_ stock solution with varying gadobutrol concentrations ranging from 1 to 40 mM and baseline T1 relaxation time comparable to human blood).

### Pharmacokinetic model analysis

Voxel-wise pharmacokinetic modelling was applied to the contrast agent concentration time-curves in brain tissue and blood plasma. The most parsimonious model still giving a proper fit to the data was used, namely the Patlak model, which assumes no reflux from the brain tissue back to the blood and has been demonstrated to be suitable for brain tissue [63].

Applying the Patlak model to the contrast concentration time-curves gave us an estimation of the *K*_*i*_-parameter (min^−1^) and the *v*_*p*_-parameter for each voxel. The *K*_*i*_ rate can be considered a measure of the permeability surface area product that indicates the leakage rate from the blood plasma to the brain tissue, and the *v*_*p*_-parameter can be considered a measure of the blood plasma volume. Histograms of these parameters in the grey and white matter and hippocampus were created and corrected for noise [15], after which the mean *K*_*i*_ and *v*_*p*_ were calculated for each region. Due to the noise correction, mean *K*_*i*_ per ROI generally has a lower value than the leakage rate of specific voxels (as depicted on overlays) as a relatively large number of voxels do not exhibit obvious leakage or leakage levels below the noise level.

### Statistics

Among the cognitive scores, only Stroop interference was non-normally distributed. Difference score and current score of Stroop interference and the leakage rate and blood plasma volume parameters were cube-root transformed to obtain normal distributions. WMH volume and hippocampal volume were divided by intracranial volume to correct for head size [60] and log-transformed to obtain normal distributions.

Multiple linear regression was performed, with cognitive decline as dependent variable and leakage rate as predictor, in turn for the white matter, grey matter and hippocampus separately, while correcting for age, sex and education. G*power software was used to calculate the required sample size [64]. We wanted to be able to detect a medium effect size (*f*^2^ = 0.15 [65]), with an overall significance level of 5% and power set at a conventional 80%. Power analysis for multiple linear regression using four predictors [66] demonstrated that 55 participants would be sufficient, and we eventually included 57 participants, taking into account the possibility that a few participants might have had to be excluded in the post-processing phase. The regression models were tested for each cognitive domain, resulting in a total of four times, and results were therefore corrected for multiple comparisons using the Benjamini–Hochberg procedure [67], with four tests and a false discovery rate (FDR) of 5%. Additional analyses were performed using the most recent measure of cognitive performance instead of cognitive decline, and using the blood plasma volume instead of the leakage rate.

Post hoc analyses were conducted to investigate the influence of other common indicators of structural brain integrity, namely WMH volume, cortical thickness and hippocampal volume, and blood plasma volume. These variables were separately added to the regression analyses as potential confounders for the relation between BBB leakage and cognition. Also, the association between these brain health measures and cognitive decline was investigated, using multiple linear regression with cognitive decline as dependent variable and WMH volume, cortical thickness, hippocampal volume or blood plasma volume as predictor, correcting for age, sex and education.

All statistical analyses were performed with commercial software (SPSS, version 24.0, IBM Corp., Armonk, NY, USA).

## Results

Multiple linear regression was used to investigate the association between BBB leakage rate (white matter, grey matter and hippocampus) and 12-year cognitive decline (short-term and long-term memory function, processing speed and executive functioning), in a usual ageing sample (*n* = 57, see Table 1 for sample characteristics).

**Table 1:** Participant (*n* = 57) characteristics

	Mean (standard deviation)/percentage/ median (25th–75th percentile)	
Age	65.8 (10.2)	
% Male	52.6	
% Level of education^a^	1/2/3	15.8/54.4/29.8
MMSE^b^	29.0 (28.0–30.0)	
% WMH Fazekas^c^	0/1/2/3	5.3/70.2/12.3/12.3
% GCA^d^	0/1/2/3	19.3/50.9/24.6/5.3
% MTA^e^		
Right hemisphere	0/1/2/3/4	78.9/17.5/3.5/0.0/0.0
Left hemisphere	0/1/2/3/4	78.9/19.3/1.8/0.0/0.0

^a^Level of education: 1, at most primary or lower vocational education; 2, secondary education; 3, higher vocational or scientific education

^b^Mini-Mental State Examination score [44] at 12-year follow-up: maximum score = 30, cognitively normal ≥ 25

^c^Fazekas visual rating score of white matter hyperintensity load [56]: 0 = absent: none or a single punctuate WMH lesion; 1, mild: multiple punctuate lesions; 2, moderate: beginning of confluency of lesions; 3, severe: large confluent lesions

^d^Global cortical atrophy visual rating score [57]: 0, absent: normal volume/no ventricular enlargement; 1, mild: opening of sulci/mild ventricular enlargement; 2, moderate: volume loss of gyri/moderate ventricular enlargement; 3, severe: ‘knife blade’ atrophy/severe ventricular enlargement

^e^Medial temporal lobe atrophy visual rating score [58]: 0, absent: no atrophy; 1, marginal: only widening of choroid fissure; 2, mild: also widening of temporal horn of lateral ventricle; 3, moderate: moderate loss of hippocampal volume; 4, severe: severe volume loss of hippocampus

### Cognitive decline

A significant decrease in cognitive score was found for immediate recall (*p* = .023), processing speed (*p* < .001) and interference (*p* < .001).

#### The association between BBB leakage and cognitive decline

**Fig. 1:**
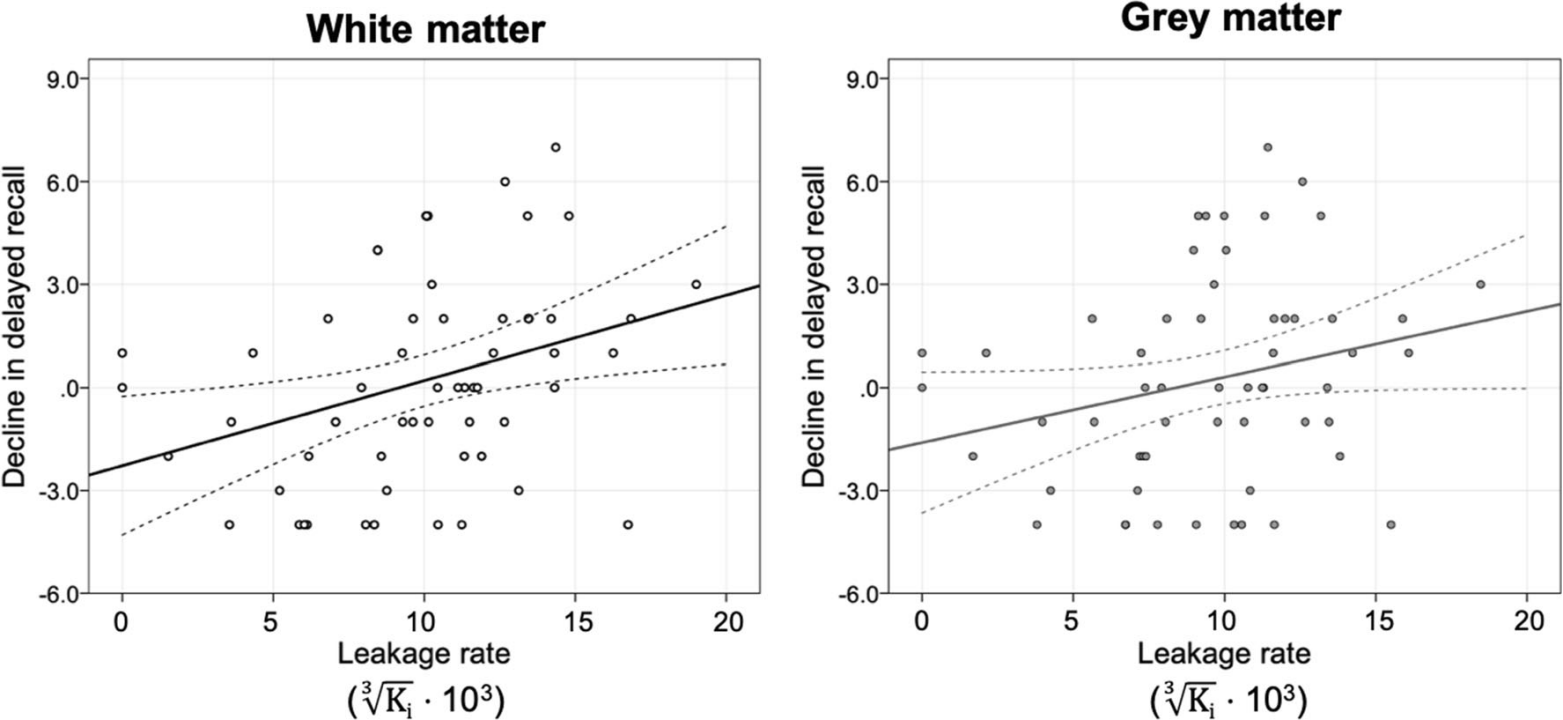
Regression analysis demonstrated that white and grey matter leakage rate is significantly associated with decline in delayed recall (*n* = 57)

**Fig. 2:**
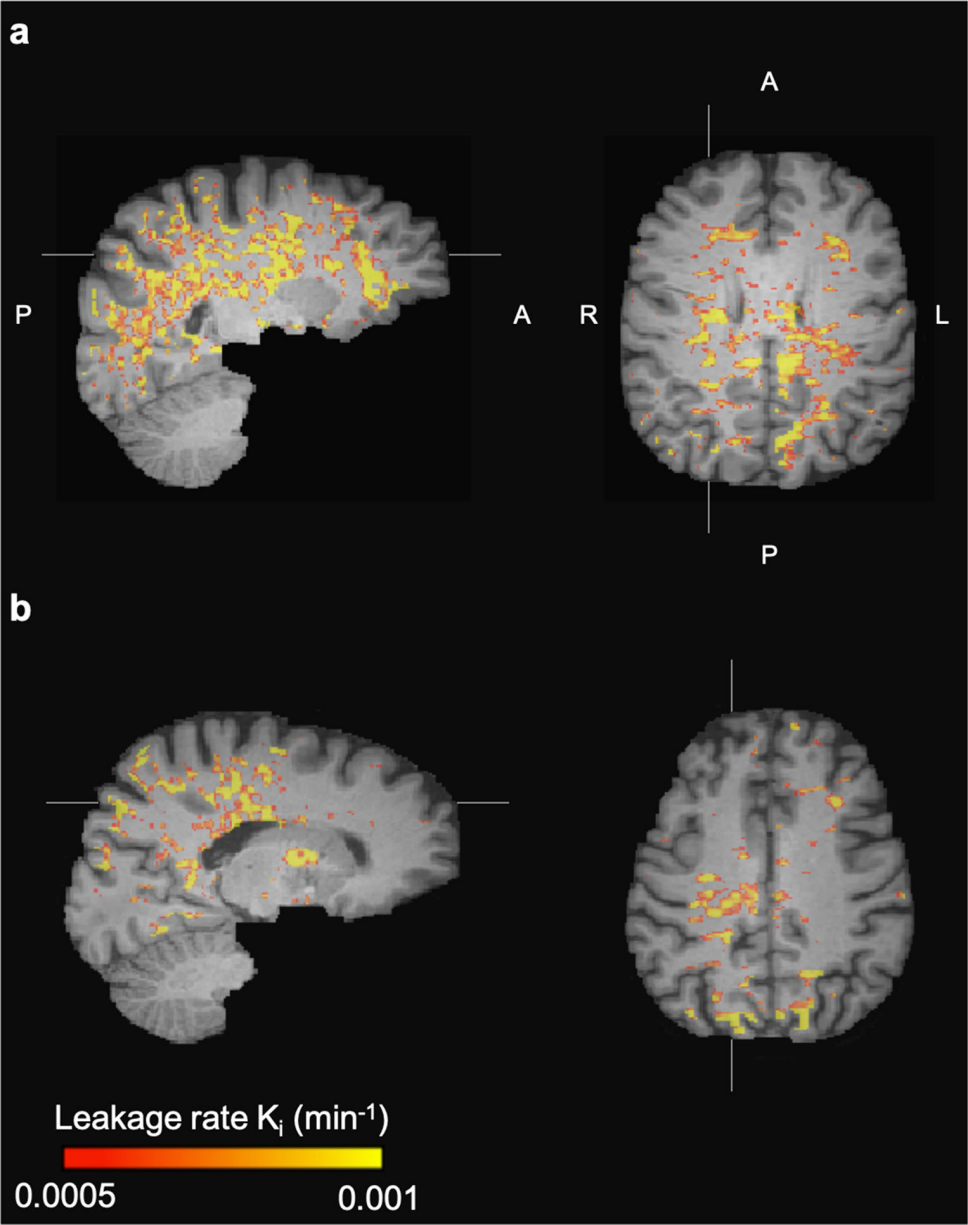
Leakage rate per voxel in the white matter of **a** a woman (57 years) with a large decrease in the delayed recall score (remembering seven words less) and **b** a man (62 years) with improved performance on the delayed recall test (remembering four words more)

Older age is significantly associated with higher BBB leakage in the white matter (*β* = .306, *p* = .024) and grey matter (*β* = .286, *p* = .035), as can be read in more detail in Verheggen et al. [50]. Upon investigating whether this higher leakage is also associated with more age-related cognitive decline, we found a significant relation between BBB leakage in the white and grey matter and decline in delayed recall (Table 2; Fig. 1; Fig. 2), with higher BBB leakage rates being associated with a larger decrease in delayed recall score, while correcting for age, sex and education (white matter *β* = .389, *p* = .006; grey matter *β* = .287, *p* = .044). Out of the four cognitive domains, leakage rate had the strongest regression coefficients with delayed recall (Table 2).

**Table 2:** Median and interquartile range (IQR) of the leakage rates and mean and standard deviation (SD) of cognitive decline in each cognitive domain, and the standardized regression coefficient (*β*) between leakage rate and cognitive decline, corrected for age, sex and education

			White matter	Grey matter	Hippocampus
		*K*_*i*_ (10^−6^ · min^−1^) Median (IQR)	1.1 (0.5; 2.0)	0.9 (0.4; 1.6)	1.7 (0.4; 4.2)
	Cognitive decline^a^ Mean (SD)				
Immediate recall	2.4 (6.6)*		*β* = .200	*β* = .083	*β* = .056
Delayed recall	0.3 (2.9)		*β* = .389*	*β* = .287*	*β* = .175
Processing speed	5.1 (4.4)*		*β* = .075	*β* = .069	*β* = .038
Interference^b^	13.5 (11.0)*		*β* = .176	*β* = .150	*β* = .054

^a^Score last MAAS measure—current score

^b^Reversed by multiplying with − 1

*Significant at *p* < .05

After correction for multiple comparisons using the Benjamini-Hochberg procedure [67] (four tests, FDR = .05), only the association between decline in delayed recall and white matter leakage remained significant.

Multiple linear regression with BBB leakage in the white matter as dependent variable and age as predictor, while correcting for sex, gave an unstandardized regression coefficient (*β*) of 1.2 · 10^−4^. This suggests that being 10 years older equals an increase of 1.2 · 10^−3^ in the (cube-root transformed) white matter *K*_*i*_ rate. The above reported regression with white matter BBB leakage as predictor of decline in delayed recall score (standardized *β* = .39 (Table 2), unstandardized *β* = 286) can be used to calculate that this amount of leakage would correspond to a decline of 0.35 units (286 · (1.2·10^−3^)) on the VLT delayed recall score. Considering the baseline MAAS data from 1855 healthy participants [68], not taking BBB leakage into account, this decline in cognition would normally correspond to an increase of 4.7 years of age. Thus, over the 10-year period, white matter BBB leakage alone is estimated to be associated with 4.7 years of cognitive ageing.

#### The association between BBB leakage and current cognitive score

No significant association was found between BBB leakage in the white or grey matter, or hippocampus, and the most recent cognitive score for each cognitive domain. However, the association between BBB leakage in the white and grey matter and delayed recall score approached significance (white matter *β* = − .259, *p* = .055; grey matter *β* = − .264, *p* = .050), showing a trend towards more BBB leakage being associated with a worse performance on the delayed recall test.

#### The association between blood plasma volume and cognitive decline

No significant association was found between blood plasma volume in the white matter, grey matter or hippocampus and decline in any of the cognitive domains (white matter *β* ≥ − .171, *p* ≥ .19; grey matter *β* ≥ − .209, *p* ≥ .11; hippocampus *β* ≤ .139, *p* ≥ .33).

Please note that the main analyses, i.e. BBB leakage and 12-year cognitive decline, was tested and corrected for multiple comparisons. No correction for multiple comparisons was applied to the analyses between BBB leakage and current cognitive score, or the analyses between blood plasma volume and cognitive decline, as these were additional analyses that yielded no significant results, and multiple comparisons correction, to check whether a significant result remains significant after correction, was not needed.

#### The influence of WMH volume, cortical thickness, hippocampal volume and blood plasma volume

Post hoc analyses demonstrated that the association between BBB leakage in the white matter and decline in delayed recall remained significant after correction for WMH volume, cortical thickness, hippocampal volume or blood plasma volume. The association between grey matter BBB leakage and decline in delayed recall was just above significance when correcting for WMH volume (*p* = .061), cortical thickness (*p* = .064), hippocampal volume (*p* = .066) or blood plasma volume (*p* = .080). Results for the analyses with other ROIs or other cognitive domains did not change when adding WMH volume, cortical thickness, hippocampal volume or blood plasma volume to the regression analyses. None of these other MRI measures of structural brain integrity or blood plasma volume had a significant relation with cognitive decline.

## Discussion

In the current study, we investigated whether BBB disruption is related to usual age-related cognitive decline. We found a significant association between BBB leakage and decrease in delayed recall score, which measures long-term episodic memory. Previous research has shown that decline in episodic memory is part of the normal ageing process [69, 70]. Both acquisition (the ability to encode newly learned information into memory) and retrieval (the ability to access newly learned information) decrease across the lifespan. The delayed recall score is considered a measure of retrieval and we demonstrated that BBB disruption in the white matter was significantly associated with impairment in retrieval. The current study estimated that over a period of 10 years, white matter BBB leakage alone would be associated with 4.7 years of cognitive ageing. BBB disruption in the grey matter also showed an association with decline in retrieval, but this was less strong than in the white matter and no longer significant after correction for multiple comparisons.

The encoding of episodic events takes place in the hippocampus [71] first, and representations of these events are then stored in the cortex. For the retrieval function, both hippocampal and cortical processing is required with activation of a hippocampal–cortical network [71, 72]. Especially pathology in the white matter tracts can disrupt communication between the hippocampus and cortex and may impair network function. This corresponds to our finding that decline in retrieval is strongly related to BBB disruption throughout the cerebral white matter, rather than specifically in the hippocampus. For future studies, it would be interesting to investigate what white matter tracts are involved and in which white matter regions the association between BBB leakage and cognitive decline is most prominent.

A previous study found a significant association between BBB leakage and age and demonstrated that BBB leakage was significantly higher in individuals with mild cognitive impairment compared to normal controls [38]. Remarkably, these relations were only found for BBB leakage in the hippocampus, and not for leakage in white matter or cortical grey matter structures. The authors propose that, in normal ageing, BBB disruption possibly begins in the hippocampus and may subsequently contribute to age-related cognitive impairment. However, while the current study demonstrated that BBB disruption was linked to usual age-related cognitive decline, we could only find this association for BBB disruption in the cerebral white matter, but not in the hippocampus. Thus, we were not able to find support for the previous suggestion that early BBB leakage beginning in the hippocampus contributes to cognitive impairment in normal ageing.

In a later study, the same group found higher BBB leakage in the hippocampus of individuals with early cognitive dysfunction compared to healthy controls [17]. Possibly, the relation between BBB disruption in the hippocampus and cognitive performance is only present at a later stage of age-related cognitive deterioration. In another more recent study, they elaborate on this finding by demonstrating that cognitive impairment could especially be linked to BBB leakage in the hippocampus among APOE4 carriers [45]. As the APOE4 allele greatly increases the risk of a neurodegenerative disorder, especially AD [40], this indicates that BBB disruption in the hippocampus might be more involved in pathological ageing and might explain why the current study, focusing on successful ageing, obtained a different result. However, another previous study, not only investigating normal controls but also individuals with mild cognitive impairment and AD, found no significant relation between BBB disruption in the hippocampus and cognitive performance [73]. The authors suggest that, at a later stage of neurodegeneration, the effect of microvascular damage on cognitive performance might be negligible compared to the effect of neurodegenerative processes. This study also did not find a significant relation between BBB disruption in the white or grey matter and memory performance. The discrepancy with our results could be due to the use of longitudinal cognitive data in our study. When using only the most recent measure of cognitive performance, we did not find a significant relation either, though a trend approaching significance was visible. This could indicate that the influence of BBB disruption on cognitive performance is best visible in the long term.

Performance on the cognitive tests is not only determined by cognitive ageing but also practice effects could influence the scores [74]. Especially memory scores are known to be susceptible to practice [75, 76]. Cognitive decline could result from a combination of more severe cognitive ageing and less benefit from practice effects, and this combination can differ for each cognitive domain. Therefore, the current study did not aim to compare cognitive decline between the different domains. Instead, we investigated whether variation in a particular cognitive score was related to variation in leakage rate.

We did not find associations between BBB leakage and decline in immediate episodic memory as a measure of acquisition, processing speed, or the interference score as a measure of inhibition. As mentioned before, acquisition has been shown to decrease across the life span [69, 70]. Also, processing speed decreases with age, starting in the third decade [69, 77], and research has shown that inhibition is compromised in normal ageing, older adults [78, 79]. Although each cognitive domain is thus known to be affected by age, only for decline in retrieval the association with BBB disruption reached significance. Previous imaging studies using diffusion tensor imaging (DTI) have suggested that usual age-related cognitive decline can at least partly be explained by white matter damage, with the greatest effect on cognitive functions that depend on communication between brain regions [80, 81]. In a comparable sample of healthy, middle-aged to older individuals, no direct association could be found between white matter integrity, as measured with DTI, and processing speed or executive function [81]. However, white matter integrity was significantly associated with working memory, possibly because working memory involves communication between the medial temporal and the frontal system [80, 82]. Already before these findings, working memory had been linked to early normal ageing, which was attributed to a disruption in balance between the two systems [83]. As mentioned above, retrieval also involves communication between medial temporal lobe structures and distributed cortical areas [71, 72], so this cognitive function could be most vulnerable to usual age-related decline, which would explain why we only found a significant relation with decline in retrieval.

After correction for WMH volume, cortical thickness, hippocampal volume or blood plasma volume, BBB disruption in the white matter remained significantly associated with decline in retrieval. Thus, the relation between BBB disruption and usual age-related cognitive decline could not be explained by other physiological ageing phenomena. BBB leakage being significantly associated with cognitive decline even after correction for blood plasma volume also indicates that the *K*_*i*_ rate is not simply dependent upon capillary density. No extensive white matter lesion load, cortical thinning or hippocampal volume loss was present in the current study, as can be seen in the overall low Fazekas, GCA and MTA ratings (Table 1), and these variables were not associated with cognitive ageing. Also, while decreased vascularization has been associated with cognitive decline [84, 85], our study does not find any association between the blood plasma volume and cognition. However, BBB disruption was already associated with usual age-related cognitive decline at this stage, which supports the notion that BBB disruption is an early event in cognitive ageing, that might possibly affect brain function before these other processes.

To confirm our findings, longitudinal data should be collected to investigate whether BBB leakage increases as a person ages and whether this increase is paired with a change in cognitive function. Not only will a within-subject longitudinal design have more power as the results are no longer influenced by between-subjects variation, it will also allow us to comment on causality and uncover whether BBB disruption precedes cognitive changes and other brain pathologies. With BBB leakage rate only being obtained at the end of the 12-year interval, one cannot make inferences about causal relations, as persons with stronger BBB disruption and cognitive decline may have similar underlying origins. Neuroinflammation, for example, seems to form a vicious cycle with BBB disruption [86]. Defensins penetrate the BBB and trigger the release of inflammatory cytokines, which leads to BBB disruption and consequently access of defensins, T cells and inflammatory mediators into the brain, further promoting neuroinflammation. Another example is cerebral small vessel disease (cSVD). cSVD burden was demonstrated to correlate with both stronger BBB disruption and smaller blood plasma volume [87]. Just like BBB disruption, neuroinflammation and cSVD have both been implicated in neurodegeneration and cognitive impairment [86, 88, 89], and these processes may all be interacting with one another. It would have been preferable to measure BBB leakage at the moment of the first cognitive measure, so we would have been able to investigate whether baseline BBB disruption predicts future cognitive decline. However, 12 years ago, imaging techniques were not yet sensitive enough for the subtle leakage values in normal ageing, which rendered the current design the best alternative. Moreover, BBB leakage needs to be MRI detectable at these younger ages in similarly healthy persons.

Another point of consideration for this study was the difficulty in identifying usual age-related cognitive decline. Participants recruited from MAAS have been participating in research for over 25 years, and may represent an exceptionally healthy subgroup, who are likely to perform above-average for their respective age groups. Cognitive decline in normal ageing is subtle, especially in a sample this healthy. The standard cognitive tests, especially the memory scores, can lack sensitivity due to practice effects [75, 76], even in the current study with cognitive decline being calculated over a 12-year interval. To minimize these effects, different versions of the VLT were used at the different time points (but fixed per time point), which have been demonstrated to be equivalent [90]. Moreover, this study benefitted from the use of the cognitive MAAS data [51]. Initial practice has the strongest effect [75], and we were able to use the third MAAS measure as our baseline to minimize practice effects.

## Conclusion

BBB disruption throughout the cerebral white matter was associated with decline in retrieval in normal ageing. BBB breakdown being related to early cognitive decline supports the notion that BBB disruption could be a trigger of neuropathology eventually leading to neurodegeneration, and suggests that it could be a contributing mechanism to cognitive ageing. Future research should investigate the pathological sequence of events by using longitudinal imaging data. Moreover, cardiovascular risk and life-style factors could be relevant to study possible mitigation of the observed effect. Also, recent studies suggest that restoring NAD^+^ levels can protect BBB integrity [91–93], and this effort might become relevant for the maintenance of cognitive function.

## Data Availability

The dataset supporting the current study has not been deposited in a public repository to keep participant data secure, but is available from the corresponding author on request.
